# Unequal misses during the flash-induced advancement of photosystem II: effects of the S state and acceptor side cycles

**DOI:** 10.1007/s11120-018-0574-0

**Published:** 2018-09-06

**Authors:** Long Vo Pham, Julian David Janna Olmos, Petko Chernev, Joanna Kargul, Johannes Messinger

**Affiliations:** 1 Department of Chemistry - Ångström, Uppsala University, Lägerhyddsvägen 1, 75120 Uppsala, Sweden; 2 Solar Fuels Lab, Centre of New Technologies, University of Warsaw, Banacha 2C, 02-097 Warsaw, Poland; 3 Department of Chemistry, Chemistry Biology Center (KBC), Umeå University, Linnaeus väg 6, 901 87 Umeå, Sweden; 4 Present Address: Department of Molecular Biophysics, Faculty of Biochemistry, Biophysics, and Biotechnology, Jagiellonian University, Gronostajowa 7, 30-387 Kraków, Poland

**Keywords:** Photosynthesis, Photosystem II, Mechanism of water oxidation, Flash-induced oxygen oscillation pattern (FIOP), Unequal miss parameter, *Cyanidioschyzon merolae*

## Abstract

Photosynthetic water oxidation is catalyzed by the oxygen-evolving complex (OEC) in photosystem II (PSII). This process is energetically driven by light-induced charge separation in the reaction center of PSII, which leads to a stepwise accumulation of oxidizing equivalents in the OEC (S_*i*_ states, *i* = 0–4) resulting in O_2_ evolution after each fourth flash, and to the reduction of plastoquinone to plastoquinol on the acceptor side of PSII. However, the S_*i*_-state advancement is not perfect, which according to the Kok model is described by miss-hits (misses). These may be caused by redox equilibria or kinetic limitations on the donor (OEC) or the acceptor side. In this study, we investigate the effects of individual S state transitions and of the quinone acceptor side on the miss parameter by analyzing the flash-induced oxygen evolution patterns and the S_2_, S_3_ and S_0_ state lifetimes in thylakoid samples of the extremophilic red alga *Cyanidioschyzon merolae*. The data are analyzed employing a global fit analysis and the results are compared to the data obtained previously for spinach thylakoids. These two organisms were selected, because the redox potential of *Q*_A_/*Q*_A_^−^ in PSII is significantly less negative in *C. merolae* (*E*_m_ = − 104 mV) than in spinach (*E*_m_ = − 163 mV). This significant difference in redox potential was expected to allow the disentanglement of acceptor and donor side effects on the miss parameter. Our data indicate that, at slightly acidic and neutral pH values, the *E*_m_ of *Q*_A_^−^/*Q*_A_ plays only a minor role for the miss parameter. By contrast, the increased energy gap for the backward electron transfer from *Q*_A_^−^ to Pheo slows down the charge recombination reaction with the S_3_ and S_2_ states considerably. In addition, our data support the concept that the S_2_ → S_3_ transition is the least efficient step during the oxidation of water to molecular oxygen in the Kok cycle of PSII.

## Introduction

Cyanobacteria, algae, and higher plants capture sunlight to store its energy in the chemical bonds of carbohydrates, proteins, and lipids. The electrons and protons used for atmospheric CO_2_ fixation are extracted from water. The electrons are then energized to the reduction potential required for CO_2_ reduction in two light-driven charge separation reactions performed in the reaction centers of photosystems II (PSII) and photosystem I (PSI), and stabilized by multiple electron transfer events to allow the slow chemistry of water oxidation and CO_2_ fixation to occur. The side product of solar-driven water oxidation is molecular oxygen, which sustains all aerobic life on Earth (Govindjee et al. 2005; Renger 2008; Barber 2009; Shevela et al. 2012; Blankenship 2014; Cardona et al. 2015).

**Fig. 1:**
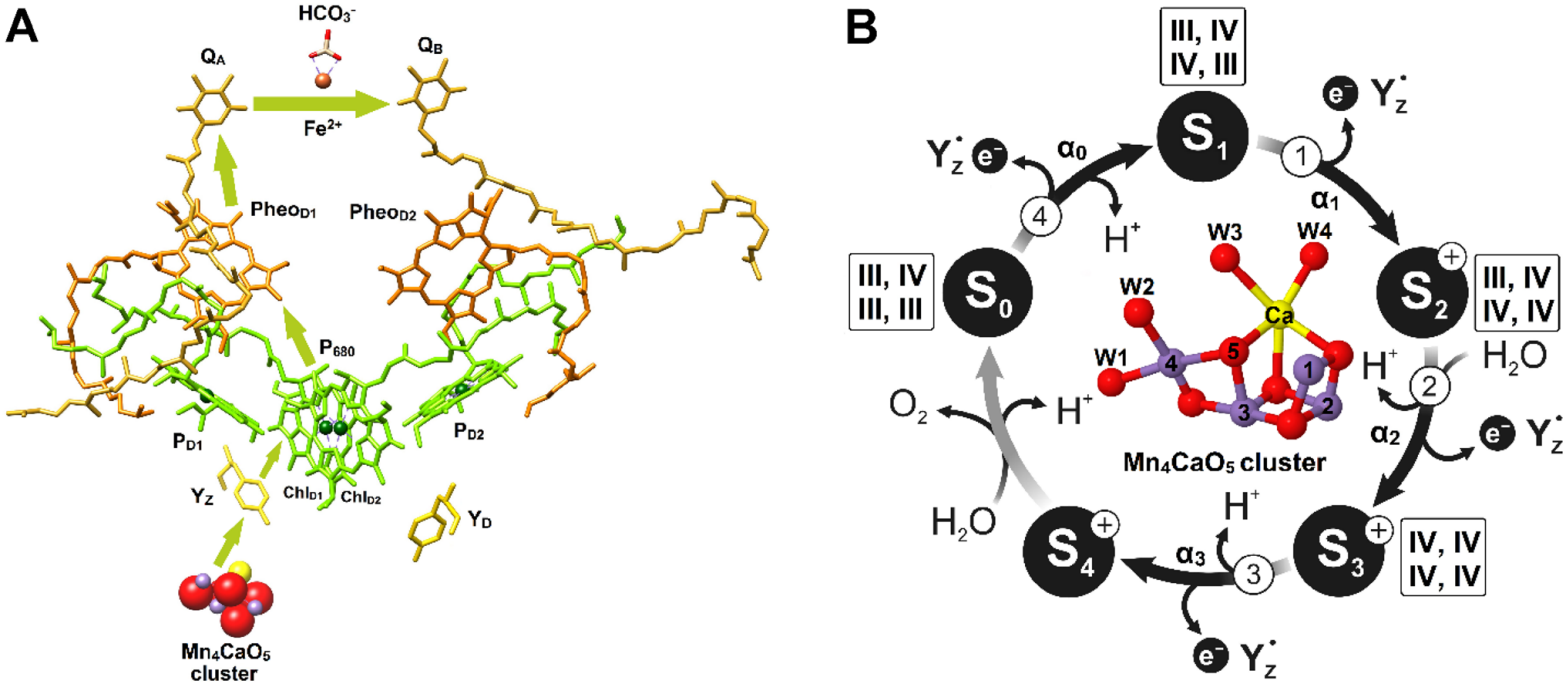
The electron transfer cofactors of the PSII reaction center (left, **a**) and the Kok cycle of solar-driven water oxidation and the structure of the Mn_4_CaO_5_ cluster (right, **b**). S_*i*_-state-dependent miss parameters, proton release, and substrate water binding are indicated. Numbers on the arrows correspond to flash numbers driving the first cycle, which starts from the dark-stable S_1_ state. The oxidation states of Mn are indicated in the order Mn1, Mn2, Mn3, Mn4 of the Mn_4_CaO_5_ cluster, which is depicted in the center of the Kok cycle (see Kok et al. 1970). α_i_ signifies the miss parameter connected to the oxidation of the respective S_*i*_ state (*i* = 0–4). Color code: O red, Mn purple, Ca yellow. W1–W4 are terminal water ligands (W2 may be a hydroxo) of Mn4 and Ca. The structures shown are based on pdb 5TIS (Young et al. 2016)

The reaction sequence in the PSII complex, which catalyzes water oxidation to dioxygen, can be divided into three main events that occur at different time scales. Photon capture in the light-harvesting antenna of PSII and excitation energy transfer to the reaction center lead to the formation of the excited state of the primary donor, P680^*^, which is a chlorophyll (Chl) dimer. P680^*^ transfers its excited electron within picoseconds to the primary acceptor pheophytin, Pheo (Fig. 1a) (Mamedov et al. 2015; Mirkovic et al. 2017; Romero et al. 2017). The subsequent electron transfer steps, which increase the spatial separation and decrease the potential difference between the photo-generated charge pair, minimize the chance for charge recombination reactions. These processes occur over a wide time span (10 ns–100 µs) and include on the acceptor side electron transfer from Pheo^•−^ to the firmly bound plastoquinone *Q*_A_, and further to the second, exchangeable plastoquinone, *Q*_B_. On the donor side, P680^•+^ is reduced by tyrosine *Z, Y*_Z_ (D1-Tyr161), which in turn oxidizes the oxygen-evolving complex (OEC) of PSII. Repetition of these steps allows for multi-electron and multi-proton chemistry to occur at the 100 µs–1 ms time scales. These reactions form plastohydroquinone on the acceptor side and molecular oxygen in the OEC on the donor side of PSII (Renger and Renger 2008; Blankenship 2014).

At the heart of the OEC is the Mn_4_CaO_5_ cluster (Fig. 1b, center). This inorganic core unfolds its full catalytic potential within the special protein and water environment of its binding pocket. The structure of this complex catalytic site has been revealed by years of research employing EPR, EXAFS, and X-ray crystallography measurements, as well as theoretical calculations for recent contributions (e.g., Messinger et al. 1997; Peloquin et al. 2000; Zouni et al. 2001; Cinco et al. 2002; Robblee et al. 2002; Ferreira et al. 2004; Yano et al. 2006; Kulik et al. 2007; Dau and Haumann 2008; Siegbahn 2009; Umena et al. 2011; Glöckner et al. 2013; Shoji et al. 2013; Cox et al. 2014; Yano and Yachandra 2014; Krewald et al. 2015; Suga et al. 2015; Young et al. 2016). This pocket positions a network of hydrogen-bonded water molecules around the inorganic unit (Umena et al. 2011), thereby orchestrating the proton-coupled electron transfer events required for the accumulation of four oxidizing equivalents and the subsequent water oxidation chemistry (Lavergne and Junge 1993; Rappaport and Lavergne 1997; Dau and Haumann 2008; Service et al. 2010; Rappaport et al. 2011; Nakamura et al. 2016; Nilsson et al. 2016).

On the basis of the period four oscillation during flash-induced oxygen evolution, first measured by Joliot et al. (1969), and additional experiments, five oxidation states of the OEC were identified and named by Kok and coworkers as the S_0_, S_1_, S_2_, S_3_, and S_4_ states (Fig. 1b) (Kok et al. 1970; Joliot and Kok 1975). For different, earlier proposals, see Joliot et al. (1969) and Mar and Govindjee (1972). Proton release occurs on all transitions, except the S_1_→S_2_ transition (Rappaport and Lavergne 1991; Schlodder and Witt 1999). This is important, to allow *Y*_Z_^•^, which has a near constant oxidation potential throughout the reaction cycle, to stepwise oxidize the Mn_4_CaO_5_ cluster. Thus, in the positively charged S_2_^+^ state, initially a proton needs to be released to allow oxidation of the neutral S_2_^n^ state to the S_3_^+^ state (Rappaport and Lavergne 1991; Klauss et al. 2012). This step is also likely coupled with the binding of one water molecule to the Mn_4_CaO_5_ cluster (Haumann et al. 2005; Suzuki et al. 2008; Siegbahn 2009; Suga et al. 2015, 2017; Capone et al. 2016; Retegan et al. 2016; Ugur et al. 2016; Kim and Debus 2017). Similarly, S_3_^n^ state needs to be formed by a proton release from S_3_^+^ before the OEC can be further oxidized and in turn form O_2_ from the two substrate ‘water’ molecules (Rappaport et al. 1994; Rappaport and Lavergne 2001; Dau and Haumann 2007; Klauss et al. 2015).

The most stable oxidation state of the OEC is the S_1_ state, in which the four Mn ions have the oxidation states III, IV, IV, III; in the order of numbering in Fig. 1b (Krewald et al. 2015). The S_2_ (III, IV, IV, IV) and S_3_ (IV, IV, IV, IV) states decay to the S_1_ state within seconds to minutes via reduction by the reduced form of tyrosine *Y*_D_^RED^ (D2-Tyr160) or recombination with *Q*_B_^−^ (Diner 1977; Rutherford et al. 1982; Robinson and Crofts 1983; Rutherford and Inoue 1984; Vermaas et al. 1984, 1988; Nugent et al. 1987). In addition, a very slow decay was reported, but the electron donor for this phase was not yet identified (Styring and Rutherford 1988). By contrast, the S_0_ state is slowly oxidized to the S_1_ state by the long-lived *Y*_D_^OX^ radical, but is stable if *Y*_D_ is reduced (Styring and Rutherford 1987; Vass and Styring 1991; Messinger and Renger 1993). The highly reactive S_4_ state oxidizes two water molecules into molecular oxygen. Through this process, four electrons are injected into the OEC, effectively resetting the system for the next cycle into the S_0_ state (III, IV, III, III).

The damping of the period four oscillation in flash-induced oxygen evolution shows that the S_*i*_ state transitions do not occur with 100% efficiency. This inefficiency is described in the Kok model mostly by the miss parameter, which gives the average probability that an S_*i*_ state does not advance, upon saturating flash illumination, into the next higher S_i+1_ state (Forbush et al. 1971; Delrieu 1974; Messinger and Renger 2008). Typical values for the S_i_-state-independent miss parameter range between 10 and 15% (Messinger and Renger 1994; Isgandarova et al. 2003). Depending on the width of the exciting flash, typically also 0–5% of double advancements (S_*i*_ to S_*i*+2_ transitions) contribute to the S state mixing with increasing flash number. Under saturating illumination, the miss parameter has been proposed to be governed by either the redox equilibria between the redox cofactors at the donor or acceptor sides, or by kinetic limitations (Renger and Hanssum 1988; Meunier and Popovic 1990; Shinkarev and Wraight 1993; Christen et al. 1999; de Wijn and van Gorkom 2002; Han et al. 2012). To minimize the possibility of kinetic limitations, we employed in this study a flash frequency of 2 Hz. In addition, we explicitly account for back reactions of the S states by our global fit analysis (Vass et al. 1990; Isgandarova et al. 2003; Pham and Messinger 2016). Thus, the efficiency of the S_*i*_ state turnovers is expected to be governed by redox equilibria between all the electron transfer cofactors of PSII. These equilibria prevent, in a random fashion, a certain percentage of centers (miss parameter α) from advancing to the next higher oxidation state S_*i*_ (i = 0, 1, 2, 3, 4) by preventing a stable charge separation. In this regard, especially the presence of *Q*_A_^−^, Pheo^−^, P680^+^, and/or *Y*_Z_^•^ at the time of flash excitation is of note, which may vary with a period two on the acceptor side and a period four on the donor side (Naber et al. 1993; Shinkarev and Wraight 1993). In addition, inefficiencies of the S_*i*_ state transitions caused by deprotonation events (Forbush et al. 1971) or structural equilibria, as that in the S_2_ state between the EPR multiline and the *g* = 4.1 state (Retegan et al. 2016), may lead to a S_*i*_ state dependence of the miss parameter. Recent work by several groups provided experimental support for the S_*i*_ state dependence of the miss parameter. While fluorescence experiments by de Wijn and van Gorkom (2002) and FTIR experiments by Takumi Noguchi’s group (Suzuki et al. 2012) suggest that the miss parameters α_*i*_ increase from α_0_ to α_3_, Stenbjörn Styring and coworkers concluded on the basis of EPR experiments that the highest miss factor is associated with the S_2_→S_3_ transition (Han et al. 2012). We have recently developed a new kinetic Kok model that allows fitting a regular 2 Hz flash-induced oxygen evolution pattern (FIOP) simultaneously with 44 FIOPs obtained during the S_2_-, S_3_-, and S_0_-state lifetime measurements (Isgandarova et al. 2003; Pham and Messinger 2016). This global analysis, which works akin to decay-associated spectral analysis, indicated that the majority of misses occurs in spinach thylakoids at neutral pH in either the S_2_→S_3_ or the S_3_→S_0_ transition. In addition to misses, a small percentage of double hits (β; S_*i*_→S_*i*+2_) is observed if light pulses of µs duration are employed.

The extremophilic red alga *Cyanidioschyzon merolae* grows in hot springs at a very low pH (0.2–4.0) and moderately high temperatures (40–56 °C) (Ciniglia et al. 2004; Ferris et al. 2005). The intracellular pH of *C. merolae* is tightly controlled metabolically and maintained in the pH range of 6.3–7.1 over the extracellular pH range of 1.5–7.5 (Zenvirth et al. 1985; Enami et al. 2010). The photosynthetic apparatus of this alga is an evolutionary intermediate of cyanobacteria and higher eukaryotic phototrophs, containing combined prokaryotic and eukaryotic structural and functional features. Both PSII and PSI complexes from this acido-thermophilic alga are characterized by unprecedented robustness across a wide range of external conditions and recently molecular mechanisms underlying such resilience to adverse conditions have been revealed including reaction center-based non-photochemical quenching in PSII, accumulation of photoprotective carotenoid zeaxanthin (in PSII and PSI), and remodeling of the external light-harvesting antenna in PSI (Krupnik et al. 2013; Haniewicz et al. 2018).

The PSII complex in *C. merolae* is reminiscent of its counterpart in cyanobacteria, but the OEC is protected by the additional fourth extrinsic subunit PsbQʹ, over and above the three proteins present in cyanobacteria, PsbV, PsbU, and PsbO (Ohta et al. 2003). The PsbQʹ subunit has been localized by electron microscopy coupled to single particle analysis (Krupnik et al. 2013) as well as recently by X-ray crystallography (Ago et al. 2016) on the lumenal side of PSII in the vicinity of the CP43 and PsbV proteins, close to the membrane plane. The function of PsbQʹ has been recently elucidated by reconstitution of this protein with the cyanobacterial PSII followed by measurement of the *Q*_A_ redox potential in thus modified complex. In these experiments, the redox potential of *Q*_A_ was shown to be positively shifted when PsbQ′ was attached to the PSII complex, likely resulting in a decrease in the amount of destructive triplet Chl species (Yamada et al. 2018) and triggering a photoprotective mechanism of direct recombination between *Q*_A_^−^ and P680^+^ in high light (Krupnik et al. 2013).

**Fig. 2:**
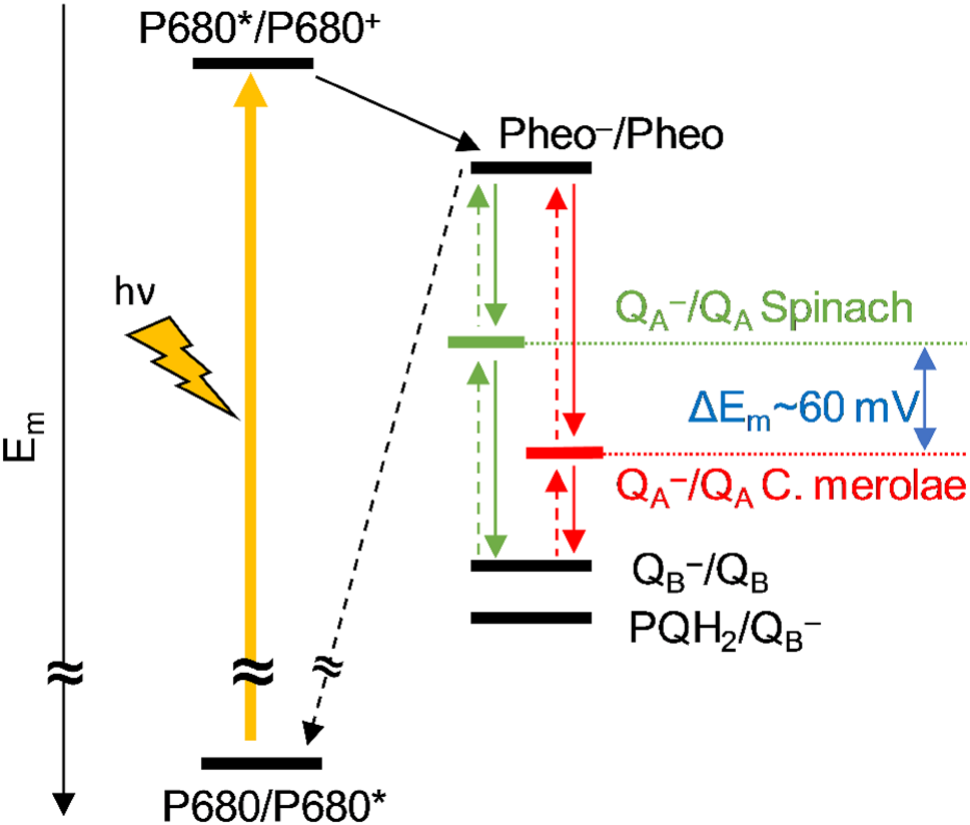
Schematic representation (not to scale) of midpoint potentials on the acceptor side of PSII in *C. merolae* and spinach. The focus is on the *E*_m_ values of *Q*_A_^−^/*Q*_A_ in both organisms, since a direct comparison has been made in one study (Shibamoto et al. 2010). We emphasize that the *E*_m_ values of the other components may also differ between the two organisms, but no comparative data were found

On the acceptor side, the potential of *Q*_A_^−^/*Q*_A_ is significantly less negative in *C. merolae* (*E*_m_ = − 104 mV) than in spinach (*E*_m_ = − 163 mV) (Shibamoto et al. 2010). Given that the midpoint potentials of the other redox cofactors, including *Q*_B_^−^/*Q*_B_, *Q*_B_H_2_/*Q*_B_^−^ and Pheo^−^/Pheo are similar in these organisms (no comparable data available in the literature), the less negative *E*_m_ of *Q*_A_^−^/*Q*_A_ is expected to increase the *Q*_A_^−^ population and thereby the miss parameter in comparison to spinach PSII centers. In addition, the larger energy gap between Pheo^−^/Pheo and *Q*_A_^−^/*Q*_A_ is expected to increase the lifetime of the S_2_ and S_3_ states in *C. merolae* PSII centers (Fig. 2). Interestingly, Allakhverdiev and coworkers reported a shift of redox potentials of Q_A_ and Pheo depending on the presence or absence of extrinsic proteins in the cyanobacterial PSII complexes of *Synechocystis* sp. and *Acaryochloris marina* (Allakhverdiev et al. 2010, 2011). Yet, the energetics of the water-splitting reaction in PSII RC was conserved, even though the potentials of *Q*_A_^−^ and Pheo^−^ were relatively shifted depending on the identity of the primary donor special pair (being Chl *a* in *Synechocystis* and Chl *d* in *Acaryochloris*), pointing towards high conservation of the electron transfer processes in oxygenic photosynthesis.

To analyze the effects of the altered *Q*_A_^−^/*Q*_A_ midpoint potential in detail and to separate it from S_*i*_-state-dependent effects, we measured flash-induced oxygen yield patterns of *C. merolae* thylakoids at three different pH values and after H/D exchange, and analyzed the data using our global fit analysis approach. High pH is expected to simplify deprotonation reactions needed to advance the Mn_4_CaO_5_ cluster into the next S_*i*_ state and to thereby reduce the donor side misses. It may also be expected that high pH makes it more difficult to protonate *Q*_B_^2−^/*Q*_B_^−^. This, in turn, may increase their midpoint potentials, likely leading to increased acceptor side misses. The results obtained in the present study are compared to literature data for spinach thylakoids measured under similar conditions.

## Materials and methods

### Thylakoid preparation

*Cyanidioschyzon merolae* cells were cultured at 42 °C and the thylakoids were isolated as described by (Krupnik et al. 2013), frozen in liquid nitrogen and stored at − 80 °C until used. On the day of the FIOP experiments, the thylakoids were thawed in the dark on ice and washed three times with buffer containing 10 mM CaCl_2_, 5 mM MgCl_2_, 1 M betaine, and either 40 mM succinic acid/NaOH (pH 5.0), 40 mM MES/NaOH (pH 6.1), or 40 mM HEPES/NaOH (pH 8.0). In the case of D_2_O experiments, the buffer was prepared in 99.9% D_2_O at 40 mM MES/NaOD (pD 6.1). In the last step, the samples were diluted to [Chl] = 0.5 mg/ml. After this treatment, which required ~ 10 min, the relative flash-induced O_2_ yields were 25% (pH 5.0), 100% (pH 6.1), and 45% (pH 8.0). No further decline in the activity was observed during the experiments. The *C. merolae* thylakoid sample had a small population of *Y*_D_^RED^ (≈ 10%). Therefore, the dark-adapted thylakoid sample was used without preflash treatment.

### FIOPs and S_*i*_-state lifetime measurements

The FIOP measurements on *C. merolae* thylakoids were performed with an unmodulated home-built Joliot-type electrode at 20 °C without adding artificial electron acceptors (Joliot 1972; Messinger and Renger 1993). Stock solutions of catalase from bovine liver (3809 U/mg, Sigma-Aldrich) were prepared by dissolving the frozen catalase powder in the respective measuring buffer. The catalase concentrations were determined using the Bradford assay (Bradford 1976; Sedmak and Grossberg 1977) and the enzymatic activities were measured using Beers assay (Beers and Sizer 1952; Aebi 1984). Then, 10,000 U/ml catalase was added into the thylakoid samples and the mixture was incubated for 10 min. In addition, the measuring buffer in the reservoir of the Joliot-electrode was continuously bubbled with N_2_ to remove O_2_ and prevent electrochemical formation of H_2_O_2_ during measurements (Pham and Messinger 2014).

For each FIOP, a fresh 10-µl aliquot of the thylakoid sample was transferred to the surface of a bare Pt-cathode in dim green light and the sample was incubated for 3 min on the electrode to allow for settling and temperature adaptation. The polarization voltage of − 750 mV was switched on 40 s before illuminating the sample with a series of 16 flashes (2 Hz; Perkin Elmer, LS-1130-4 flash lamp). A personal computer was employed to trigger the flash lamp and to record the data at a sampling rate of 3600 points/s. The S_2_-, S_3_-, and S_0_-state lifetimes of thylakoid samples were measured in the same way by exciting dark-adapted samples with one (S_2_ state formation), two (S_3_ state formation), or three (S_0_ state formation) pre-flash(es) while resting on the electrode surface. After the desired dark time (*t*_d_), a series of 16 flashes (2 Hz) was applied. The polarization was always applied 40 s before the flash series.

### Data analysis of FIOPs

**Fig. 3:**
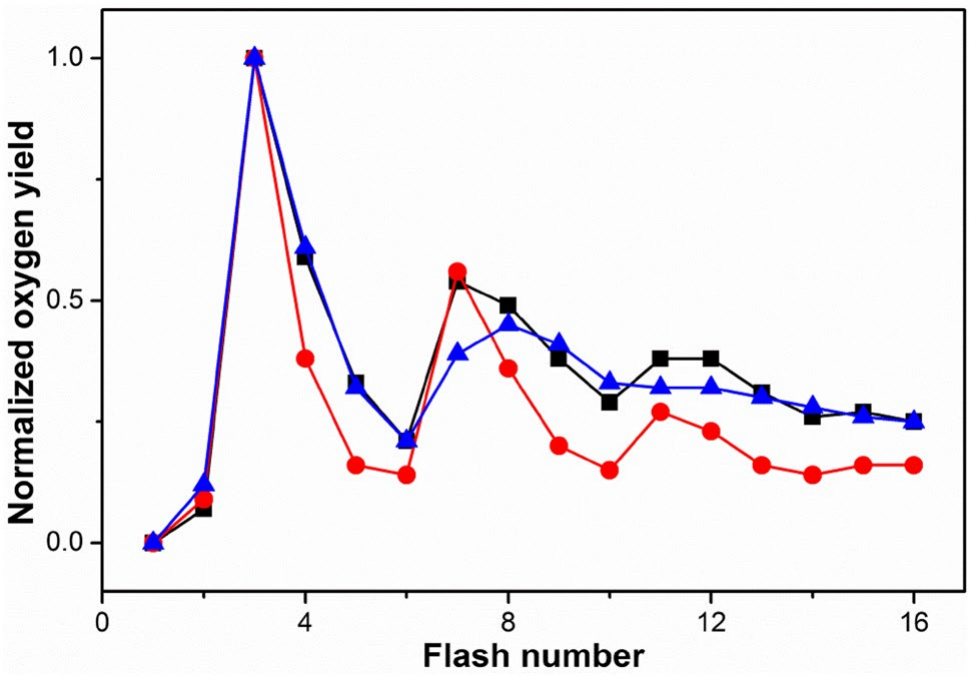
FIOPs of dark-adapted *C. merolae* thylakoids obtained at 20 °C and pH 5.0 (black squares), 6.1 (red dots), or pH 8.0 (blue triangles). The data were normalized to the oxygen yield induced by the 3rd flash. The flash frequency was 2 Hz

For each FIOP, 16 oxygen yields were recorded. However, only the first 8 flash-induced oxygen yields were used for data analysis since thereafter, a strong decline of the O_2_ yield was observed (Fig. 3). In the global fitting approach, 384 flash-induced oxygen yields comprising all time points of the S_2_, S_3_, and S_0_ lifetime measurements were directly and simultaneously fitted by adjusting the rate constants of all S_*i*_ state decays, the S_*i*_-state-dependent miss parameters, the double-hit probability, the activity/damping parameter, and the initial percentages of S_*i*_ states, *Y*_D_ and *Q*_B_^−^ (Pham and Messinger 2016).

## Results and discussion

**Fig. 4:**
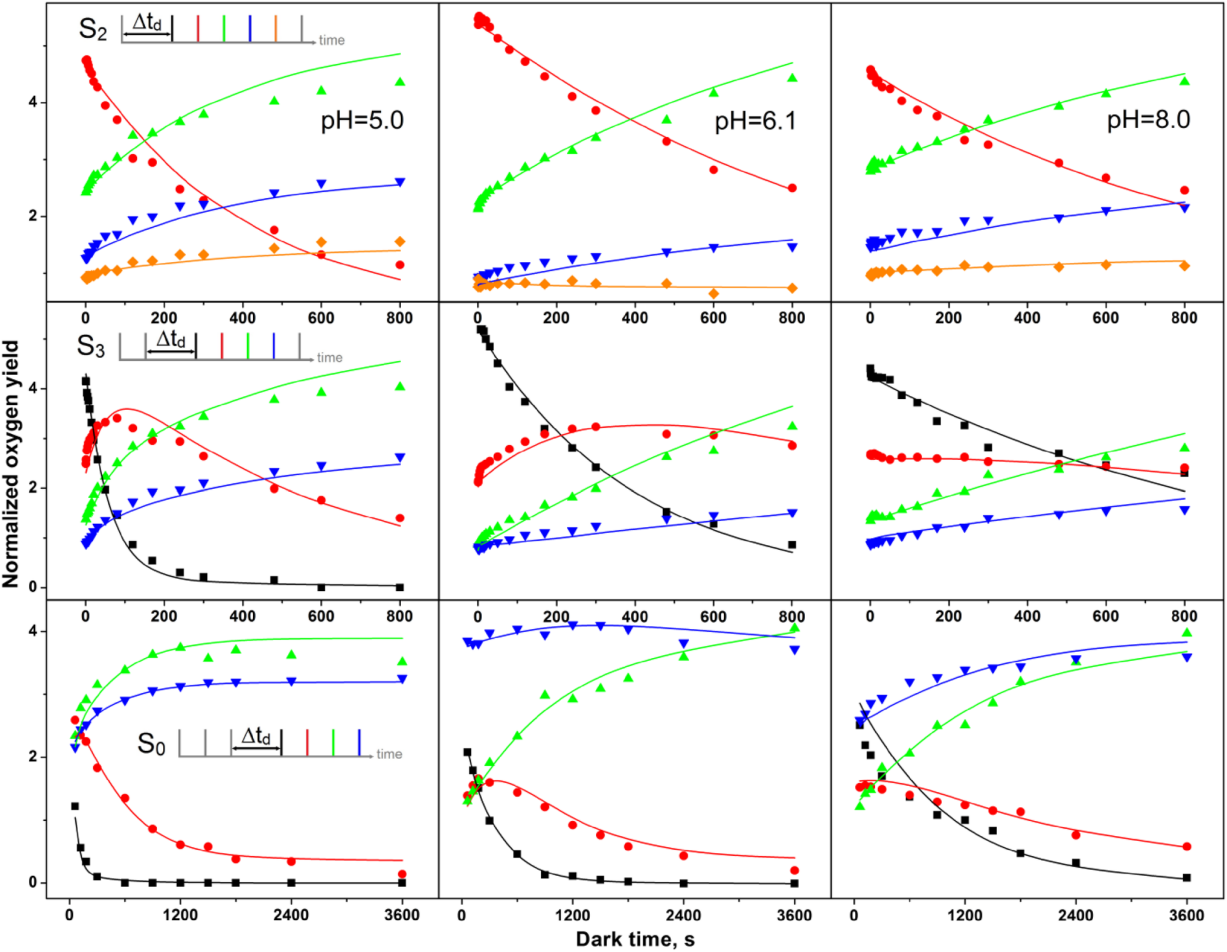
S_2_-, S_3_-, and S_0_-state lifetime measurements at pH 5.0, pH 6.1, and 8.0. The normalized flash-induced oxygen yields of flashes 2–5 (S_2_) or 1–4 (S_3_, S_0_) are plotted as a function of dark time between the pre-flash(es) and the flash train. The color code is given in the respective inserts. Symbols are the experimental amplitudes, while the lines connect the amplitudes calculated by the global fitting program. Rate constants are given in Table 1, unequal miss fit

The flash-induced oxygen evolution patterns (FIOPs) and the S_*i*_-state lifetime measurements of *C. merolae* thylakoids were recorded at pH 5.0, 6.1, and 8.0 with a Joliot-type bare platinum electrode (Isgandarova et al. 2003). The results are presented in Figs. 3 and 4, as well as in Tables 1 and 2.

**Table 1:** Fit parameters obtained during a global fit of a 2 Hz FIOP and S_3_, S_2_, and S_0_ lifetime measurements of *C. merolae* thylakoids at 20 °C and the indicated pH values

	Equal miss			S_*i*_-state-dependent miss (best fit)		
pH	5.0	6.1	8.0	5.0	6.1	8.0
α_0_, %	18	11	22	0	0	40
α_1_, %	18	11	22	0	0	0
α_2_, %	18	11	22	54	35	48
α_3_, %	18	11	22	0	0	6
β, %	1.6	3.8	3.8	1.6	2.5	2.7
d, %	98	96	96	97	95	98
*k*_32(fast)_, s^−1^	*2.0* ^a^	*1.5*	*0.028*	*3.2*	*0.32*	*4.4*
k_32(slow)_, s^−1^	0.0172	0.0030	0.0012	0.0165	0.0029	0.0012
*k*_21(fast)_, s^−1^	*1.4*	*2.1*	*0.07*	*0.8*	*1.4*	*2.4*
*k*_21(slow)_, s^− 1^	*0.0030*	*0.0015*	*0.0014*	*0.0022*	*0.0012*	*0.0012*
k_01_, s^−1^	2.3	0.0004	0.00007	2.0	0.00020	0.035
Fit quality (*10^−8^)	1625	970	600	615	495	362

Fits assuming either S_*i*_-state-independent (equal) or S_*i*_-state-dependent (unequal) miss probabilities are presented. Parameters: α_*i*_, miss parameter connected to the oxidation of the S_*i*_ state (*i* = 0–4); β double-hit parameter; d, damping/activity parameter; k_32_, k_21_, and k_01_, rate constants of S_3_, S_2_, and S_0_ decay to the S_1_ state. All fits are based on the following properties of the dark-adapted sample: 100% S_1_ state population, *Y*_D_ = 10%. A smaller value for the fit quality indicates a better fit. For further details see (Pham and Messinger 2016)

^a^Rates for the fast S_2_ and S_3_ decay, as well as for S_0_ oxidation are less reliable due to the small *Y*_D_ population giving insufficient constraints for fitting the fast S_2_ and S_3_ decays; they are thus displayed in italics

**Table 2:** Alternative fit parameters obtained during a global fit of a 2 Hz FIOP and S_3_, S_2_, and S_0_ lifetime measurements of *C. merolae* thylakoids at 20 °C and indicated pH values

pH	5.0	5.0	6.1	6.1	8.0	8.0	8.0
α_0_, %	0	0	0	0	0	0	43
α_1_, %	0	54	0	35	0	0	0
α_2_, %	0	0	0	0	57	47	0
α_3_, %	54	0	35	0	0	20	51
β, %	1.6	1.6	2.9	2.9	1.4	2.1	3.1
d, %	97	98	95	95	95	95	98
*k*_32(fast)_, s^−1^	*2.5* ^a^	*1.8*	*0.15*	*0.33*	*0.017*	*0.023*	*6.9*
k_32(slow)_, s^−1^	0.016	0.011	0.0029	0.0027	0.0012	0.0011	0.0012
*k*_*21(fast)*_, s^− 1^	0.0024*	*0.0037*	*1.2*	*1.5*	*2.3*	*0.028*	10*
k_21(slow)_, s^−1^	0.0024	0.0037	0.0013	0.0016	0.0011	0.0011	0.0015
*k*_01_, s^−1^	*2.0*	*2.0*	*0.00020*	*0.00016*	*0.00009*	*0.00090*	*0.01200*
Fit quality (× 10^−8^)	675	1330	536	645	890	600	610

The asterisk indicates that a parameter was constrained

^a^Rates for the fast S_2_ and S_3_ decay, as well as for S_0_ oxidation are less reliable due to the small *Y*_D_ population giving insufficient constraints for fitting the fast S_2_ and S_3_ decays; they are thus displayed in italics

### pH dependence of the miss parameters

Since the thylakoid preparation of *C. merolae* contained only about 10% of reduced Y_D_, the FIOPs shown in Fig. 3 can be discussed qualitatively without considering the back reactions in the dark periods of 500 ms between the flashes. At pH 6.1, a clear period four oscillation with maxima after the 3rd, 7th, and 11th flash was seen, indicating a low average miss parameter (red spheres in Fig. 3). The overall amplitude of the 3rd oscillation (flashes 11 and onwards) was, however, untypically low. This FIOP was reminiscent to that of spinach BBY preparations, indicating a limited pool of plastoquinone bound on the acceptor side of *C. merolae* PSII complex embedded in thylakoid membrane fragments.

After transferring the thylakoid samples to pH 5, a FIOP with a clearly increased miss parameter was obtained (black squares in Fig. 3). This was visible by the decreased ratio of the O_2_ yields induced by the 3rd flash (Y_3_) to that of the 4th flash (Y_4_). Similarly, the ratios Y_7_/Y_8_ and Y_11_/Y_12_ were diminished compared to those obtained at pH 6.1. Interestingly, the average O_2_ yield per flash obtained at higher flash numbers did not decrease to the same extent as at pH 6.1. This can be explained by the finding that the total O_2_ yield was only about 25% of that at neutral pH. The reduced number of active PSII complexes thus increased the number of plastoquinone molecules available for the active PSII complexes. The strong decline in the number of active PSII centers in thylakoids of *C. merolae* was surprising, since a recent report showed that the O_2_ rates of PSII core complexes isolated from the same organism are nearly invariant between pH 5.0 and 8.0 (Krupnik et al. 2013).

Comparison of the FIOP obtained at pH 8.0 (blue triangles in Fig. 3) and pH 5.0 showed that while the first 6 normalized O_2_ yields were nearly identical, clear deviations were seen thereafter. Specifically, the Y_7_/Y_8_ ratio was inverted. This was indicative of a significantly different flash number or S_*i*_ state dependence of the miss parameters at these two pH values. The total O_2_ yield was about 45% at pH 8.0 as compared to pH 6.1. This again resulted in a larger plastoquinone pool per active PSII complex.

The global fit program (GFP) analysis confirms these qualitative observations. Table 1 compares the best-fit parameters obtained at each pH by either enforcing equal misses or by allowing the miss parameter to be different for each S_i_ state transition. At pH 6.1, an equal miss fit results in a miss parameter α of about 11%, while in the best S_i_-state-dependent miss fit, all misses (35%) occur in the S_2_→S_3_ transition. Importantly, the fit quality of the unequal miss fit is twice as good as that of the equal miss fit, strongly favoring the fit where α_2_ parameter is largest. This is further supported by the finding that this fit result is systematically found once the equal miss constraint is released. However, when fits were started with a high value for α_1_ or α_3_ (all other α_*i*_ = 0), stable solutions were obtained with 35% miss in the respective S_*i*_ state transition. The fit qualities (*F*_Q_) of these fits were in between the best fit and the equal miss fit (Table 2). Nevertheless, as soon as a few percent α_2_ was included into the starting conditions, the GFP finds the solution where α_2_ = 35%. Forcing most misses into α_0_ resulted in unsatisfactory fits.

The finding that only one S state transition is responsible for all misses, whereas the other transitions occur at 100% efficiency, is counterintuitive and may be explained by shallow fit minima. Increasing manually the percentages of misses in other transitions demonstrates that the fit quality deteriorates by less than 15% if either α1 or α3 is increased up to 5% at the expense of α2. In contrast, redistributing 10% miss from α2 to α1 or α3, individually or combined, or increasing α0 by only 3% leads to a 40% worsening of the fit quality.

The fits for the pH 5.0 FIOP show the identical trends, except that the misses were higher: 18% for equal miss fits and 54% for the S_2_→S_3_ transition (Table 1). At this pH value, the unequal miss fit was nearly three times better compared to the equal miss fit. The fit where α_3_ is dominating has a nearly as good *F*_Q_ as the one with α_2_ = 54%; however, the value of k_21(fast)_ equals k_21(slow)_, i.e., it was hitting the lower fit limit (Tables 1, 2). The fit where α_1_ is forced to be maximum, results in a fit that is nearly as poor as the equal miss fit. The large difference between the equal and S_*i*_-state-dependent miss approaches comes from the—relative to Y_3_/Y_4_—large Y_7_/Y_8_ ratio, which can only be modeled correctly with unequal misses, where α_2_ or α_3_ dominates.

In contrast to pH 5.0 and 6.1, the FIOP obtained at pH 8.0 could not be fitted well when we assumed that all the misses occur in the S_2_→S_3_ transition or the S_3_→S_0_ transition (Tables 1, 2). For pH 8.0, an almost equally large percentage of misses occurred in the S_0_→S_1_ transition. Several good fits were also found where in addition to α_0_ and α_2_ also 5–20% α_1_ or α_3_ were included.

Therefore, the quantitative GFP analysis of the *C. merolae* FIOPs obtained at three different pH values strongly supports unequal misses, whereby the majority of the misses occur in the S_2_→S_3_ or, less likely, in the S_3_→S_0_ transition at acidic and neutral pH, while at alkaline pH the S_0_→S_1_ transition becomes also inefficient. If proton release was the limiting factor for the efficiency of a S_i_ state transition, one would expect the miss probability to decrease with increasing pH. While this trend was observed for α_2_ when comparing pH 5.0 and 6.1, this trend was not detected at pH 8.0.

Importantly, no binary oscillation of the miss parameter with the flash number (S_*i*_ state) was found when analyzing the pH 5.0 and 6.1 data. This supports the above analysis, which relied on the assumption that the contributions of the donor side dominate the miss parameter, while effects of the acceptor on the miss parameter are negligible.

In contrast, a pronounced binary oscillation of the miss parameter was observed at pH 8.0. This may be explained either by a high α_0_ and α_2_ or by high miss parameter connected to the presence of (*Q*_A_*Q*_B_)^−^ on the acceptor side (Naber et al. 1993; Shinkarev and Wraight 1993).

The pH jump experiments by Zaharieva et al. (2011) with spinach PSII membrane fragments, found effective pK values of 3.3, 3.5, and 4.6 for the inhibition of the S_1_→S_2_, S_2_→S_3_, and S_3_→S_0_ transitions at low pH, respectively. In contrast, none of the transitions showed decreased efficiencies at alkaline pH values. Furthermore, the S_0_→S_1_ transition was found to be pH independent between pH 3 and 9. On that basis, one would expect that the S_3_→S_0_ transition should be the only one affected by the pH under our experimental conditions. However, while the fits with large α_3_ generally gave acceptable results, they were consistently inferior to those with large α_2_. We thus suggest that the different time scales of incubation at the indicated pH, 1.5 s. vs > 10 min., are responsible for the deviating results. We also note that Zaharieva et al. (2011) observed relative changes in the efficiency of a specific S_*i*_ state transition caused by pH changes, not in the absolute α_*i*_ values, and that pH effects on the acceptor side are minimized in that study by giving only one flash at the desired pH value.

Our preferred assignment for the acidic to neutral pH (α_2_ >> α_3_, α_1_, α_0_) agrees well with the results of Han et al. (2012) who reported a similar trend by following the S_*i*_ state populations during a flash train via S_*i*_-state-specific EPR signals. A high α_2_ may be consistent with the proposed need of PSII samples in the S_2_ multiline (‘open cube’) configuration to first convert into the S_2_*g* = 4.1 state (‘closed cube’) configuration, before they can reach the S_3_ state (Retegan et al. 2016). Other possible explanations for why the highest miss parameter is connected to the S_2_→S_3_ transition may include the need to bind one additional substrate (possibly in the form of hydroxide) and the small driving force for this transition (Suzuki et al. 2008; Cox et al. 2014; Kim and Debus 2017).

The increase of the miss probability of the S_0_→S_1_ and S_2_→S_3_ transitions in the alkaline pH cannot be easily explained by any of the above proposals, unless a second independent counter effect is brought into play. Since no artificial electron acceptors were used in this study, the period of two oscillations of the miss parameter may be taken as indication that the protonation of Q_B_^2−^ became a limiting factor between pH 7 and 8, or that the *Q*_A_^−^/*Q*_A_ or *Q*_B_^−^/*Q*_B_ midpoint potentials get more similar under these conditions, leading to a high *Q*_A_^−^ population on every second flash. Further studies will be necessary to untangle the *C. merolae* PSII donor and acceptor side contributions at alkaline pH.

### pH dependence of S_*i*_-state lifetimes

For S_*i*_-state lifetime experiments, the dark-adapted thylakoid sample (S_1_ state) was excited by 1, 2, or 3 flashes to enrich PSII in the S_2_, S_3_, or S_0_ state, respectively. Then, the dark time, *t*_d_, to the subsequent FIOP flash train (2 Hz) was varied in suitable steps to probe the fast and slow decay components (see insets in Fig. 4). The symbols in Fig. 4 show how the experimental flash-induced O_2_ yields varied as a function of t_d_ during the three lifetime measurements. Plotted are either the O_2_-yields induced by flashes two to five (Y_2_–Y_5_; S_2_ state decay) or Y_1_–Y_4_ (S_3_ and S_0_ state decay). The S_2_ population can be qualitatively followed by the decay of Y_2_ (red dots), while Y_1_ tracks the S_3_ state population (black squares). The changes in the S_1_ and S_0_ populations can be estimated by amplitude changes of Y_3_ (green triangles) and Y_4_ (blue inverted triangles), respectively.

In the top row of Fig. 4, the pH dependence of the reaction S_2_→S_1_ was studied. The simultaneous decay of Y_2_ and the corresponding rise of Y_3_ demonstrated for all three pH values a clean one-electron reduction process. Due to misses and double hits, a small rise of Y_4_ and Y_5_ was also observed. Only a very small fast phase was discernible, which was followed by one slower kinetic phase. While there were not enough points for the fast phase to reliably analyze its pH dependence, it could be observed that the slow phase of S_2_ decay was about 2–3-fold faster at pH 5.0 than at the two other pH values.

The pH dependence of the S_3_ decay is best followed by the data presented in the central row. Due to the rapid S_3_ decay at pH 5.0, the sequential decay of S_3_ (Y_1_)→S_2_ (Y_2_)→S_1_ (Y_3_) could be clearly discerned. Y_4_ increased nearly in parallel to Y_3_ due to misses. For the S_3_ decay, a strong retardation of the slow decay was observed at the higher pH values. For the S_0_→S_1_ reaction, a qualitative analysis was complicated, as the expected decrease of Y_4_ was compensated by the contribution of O_2_ to Y_4_, which comes from centers in the S_1_ state that due to α1 are delayed by one flash in O_2_ production. This time-dependent increase in the S_1_ state population is caused by S_0_ oxidation and by back reactions from the S_3_ and S_2_ states. In addition, the increasing Y_D_ population further increases Y_4_ by fast reduction of fractions of S_2_ and S_3_ between the flashes of the FIOP. Nevertheless, it appears that the S_0_ oxidation exhibits a complicated pH dependence in the *C. merolae* PSII complex, with the slowest rate near neutral pH. To our knowledge, no previous data for the S_0_→S_1_ reaction had been published at pH 5 or pH 8.

The lines in Fig. 4 connect the amplitudes calculated by the GFP using the parameters given in Table 1 for the best fits. Overall, despite some small deviations, a satisfactory agreement with the data were achieved. The fits confirm the trends discussed above for the slow S_2_ and S_3_ state decays at the three pH values studied. While the S_2_ state decay is nearly pH independent (within a factor of 2), the rate of S_3_ decay slowed by more than a factor of 10 between pH 5 and 8. This is consistent with the previous data obtained from spinach thylakoids (Messinger and Renger 1994).

The most surprising finding is the very strong pH dependence of the rates for the S_0_ → S_1_ transition. While the rate at pH 6.1 was well reproduced under all tested fit conditions and agreed within a factor of four with previous estimates in spinach thylakoids (Messinger and Renger 1994) and was practically identical with that determined with *T. elongatus* thylakoids (Messinger and Renger 1994; Isgandarova et al. 2003), the much faster oxidation rates at pH 5 and 8 were surprising. Since in the present experiments we were unable to obtain strong constraints for the fast decay kinetics, the values for *k*_01_ varied at the two extreme pH values quite considerably depending on the fit scenario. At pH 5.0, values between 0.21 and 2.7 s^−1^ were obtained, while *k*_01_ was found to be in the range of 0.00007 and 0.035 s^−1^ at pH 8.0. Thus, further studies are needed to determine the precise extent of the destabilization of S_0_ at pH 5.0 and 8.0. Possible reasons for the faster S_0_ oxidation could be related to the described two different positions of a water molecule near *Y*_D_ that appear to influence the redox potential of *Y*_D_/*Y*_D_^OX^ (Umena et al. 2011; Saito et al. 2013; Sjöholm et al. 2017), and other pathways for S_0_ to S_1_ conversion may be possible under extreme pH conditions. At alkaline pH, also the easier removal of a proton may increase the rate of its conversion, since the S_0_→S_1_ transition is coupled to a proton release (Rappaport and Lavergne 1991; Siegbahn 2013; Klauss et al. 2015).

### Effects of H/D exchange

**Fig. 5:**
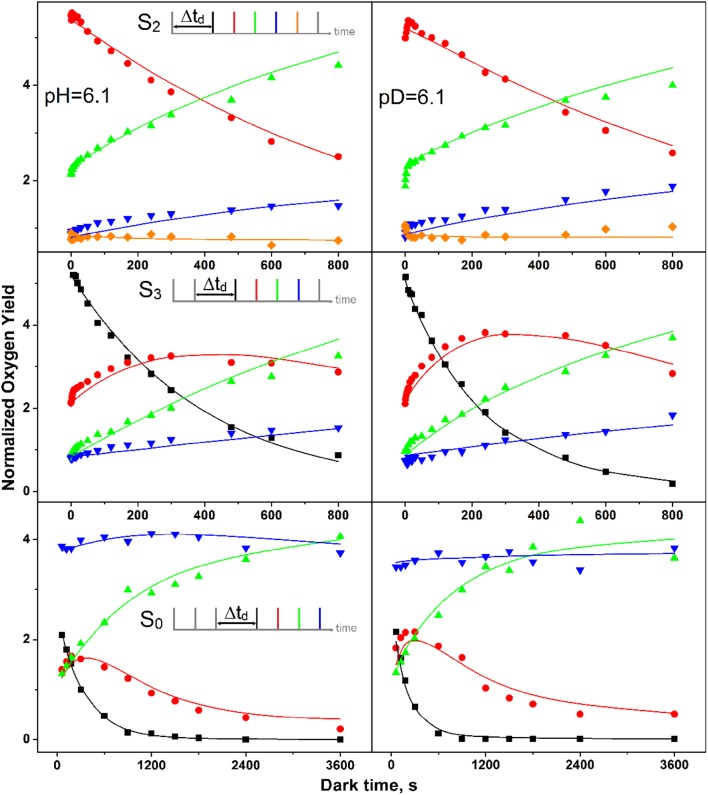
S_2_-, S_3_-, and S_0_-state lifetime measurements at pH 6.1 and pD 6.1. The normalized flash-induced oxygen yields of flashes 2–5 (S_2_) or 1–4 (S_3_, S_0_) are plotted as a function of dark time between the preflash(es) and the flash train. The color code is given in the respective inserts. Symbols are the experimental amplitudes, while the lines connect the amplitudes calculated by the global fitting program. Rate constants are given in Table 3, unequal miss fit

To further understand the above pH effects, we performed a complete GFP analysis after H/D exchange at pD 6.1 (Table 3; Fig. 5). The data in Table 3 reveal that nearly all the fit parameters are identical under H_2_O and D_2_O. However, there is a twofold increase in the rate of S_3_*Q*_B_^−^ decay [*k*_32(slow)_] and a sixfold increase of the rate in S_0_ oxidation to S_1_. In addition, a very small increase of α_2_ from 35 to 37% was observed. The slightly higher value of α_2_ in D_2_O is in line with the idea that a proton release during the S_2_→S_3_ transition is required, since a slowdown of that rate due to the greater O–D bond strength will increase the miss parameter by increasing recombination reactions between *Q*_A_^−^ and *Y*_Z_^OX^ (Zaharieva et al. 2011). However, the small magnitude of this effect indicates that this can only be one of the several factors contributing to the low efficiency of this transition.

**Table 3:** Fit parameters obtained during a global fit of a 2 Hz FIOP and S_3_, S_2_, and S_0_ lifetime measurements of non-preflashed *C. merolae* thylakoids at 20 °C and either at pD 6.1 or at pH 6.1

pL	pD 6.1	pD 6.1	pD 6.1	pH 6.1
α_0_, %	12	0	0	0
α_1_, %	12	0	0	0
α_2_, %	12	37	0	35
α_3_, %	12	0	37	0
β, %	3.8	2.6	3.0	2.5
d, %	96	96	96	95
*k*_32(fast)_, s^−1^	*0.26* ^a^	*0.10*	*0.067*	*0.32*
k_32(slow)_, s^−1^	0.0050	0.0050	0.0050	0.0029
*k*_21(fast)_, s^−1^	*2.9*	*2.8*	*2.6*	*1.4*
k_21(slow)_, s^−1^	0.0010	0.0011	0.0011	0.0012
*k*_01_, s^−1^	*0.0014*	*0.0013*	*0.0012*	*0.0002*
Fit quality (× 10^−8^)	960	575	625	495

^a^Rates for the fast S_2_ and S_3_ decay, as well as for S_0_ oxidation are less reliable due to the small Y_D_ population giving insufficient constraints for fitting the fast S_2_ and S_3_ decays; they are displayed in italics

Similarly, the acceleration of the slow S_3_ decay in D_2_O and the much shorter S_3_-state lifetimes at low pH (high proton concentration) are consistent with the need to take up a proton when returning to the S_2_ state. In line with this idea, no clear H/D effect and a much weaker pH effect were observed on the rate of S_2_ decay, which is not coupled to a proton uptake. This suggests that the pH has a strong effect on the redox potential of the S_3_ state, while the effect on *Q*_B_^−^ is less important for the S_*i*_-state lifetimes. The sixfold acceleration of the S_0_ oxidation to S_1_ by H/D exchange supports, indirectly, the surprising finding that low pH may induce the same effect (but even much stronger). The S_0_*Y*_D_^OX^ → S_1_Y_D_ reaction involves the following two half reactions:

$${S_0} \to S_{1}^{+}+{e^ - } \to {S_1}+{H^+}+{e^ - }$$

$$Y_{{\text{D}}}^{{ox}}+{H^+}+{e^ - } \to {Y_{\text{D}}}.$$

While the formation of the S_1_ state should be faster at high pH, the reduction of *Y*_D_^OX^ may occur faster at low pH. Indeed, a strong H/D effect was observed for the reactions S_2_*Y*_D_→S_1_*Y*_D_^OX^ and S_3_*Y*_D_→S_2_*Y*_D_^OX^ and was linked to the position of a water molecule near Y_D_ (Isgandarova 2004; Sjöholm et al. 2017). Zaharieva et al. (2011) observed a rapid inactivation of centers in the S_0_ state at low pH that occurs with a pK_a_ of 4.6. We thus checked our data if a specific slower phase of S_0_ state inhibition could affect the S_*i*_ state quantitation; no indications for that was found (data not shown).

### Comparison to spinach

Overall, the miss parameters and S_*i*_-state lifetimes found here for the PSII complex of the extremophilic red alga *C. merolae* were similar to those obtained earlier for spinach (Messinger and Renger 1994). This indicates that *C. merolae* PSII is not specifically adapted to acidic pH values, in agreement with earlier reports that the internal pH of *C. merolae* cells is regulated to values similar to those of higher plant chloroplasts. Consequently, the significantly different *Q*_A_^−^/*Q*_A_ midpoint potentials are not an adaptation to pH, but more likely to growth temperature. This is supported by the nearly identical rates for the slow S_2_ and S_3_ state decays in *C. merolae* and spinach if measured at 20 and 10 °C, respectively (Table 4), which demonstrates the expected stabilization of the S states by the increased energy gap between Pheo^−^/Pheo and *Q*_A_^−^/*Q*_A_. In contrast, the average miss parameter is nearly identical between these two organisms at pH 5.0 and 6.1 if measured at the same temperature (20 °C; Table 5), supporting the idea that it is determined by the unaltered donor side reactions.

**Table 4:** Fit parameters obtained by using global or single fit of one 2 Hz FIOP and/or S_3_, S_2_, and S_0_ lifetime measurements of (a) non-preflashed *C. merolae* thylakoids at 20 °C and desired pH values, (b) spinach thylakoid at 10 °C and indicated pH values

	(a) *C. merolae* 20 °C			(b) Spinach 10 °C		
pH	5.0	6.1	8.0	5.0	6.0	8.0
α, %	18	11	22	~ 12	~ 7.5	~ 9
β, %	1.6	3.8	3.8	~ 3.8	~ 2.5	~ 4
*k*_32(slow)_, s^−1^	0.0172	0.0030	0.0012	0.0173	0.0046	0.0013
*k*_21(slow)_, s^−1^	0.0030	0.0015	0.0014	0.0026	0.0020	0.0021
Reference	This study			Messinger and Renger (1994)		

**Table 5:** Fit parameters obtained during fits of 2 Hz FIOPs of preflashed spinach thylakoids (S_1_*Y*_D_^OX^) and *C. merolae* thylakoids at 20 °C and indicated pH values

	*C. merolae* 20 °C			Spinach 20 °C		
pH	5.0	6.1	8.0	5.0	6.0	8.0
α, %	18	11	22	20	12	17
β, %	1.6	3.8	3.8	2.9	2.1	2.0
Reference	This study			Pham (2011)		

## Conclusions

Global fits of the S_3_-, S_2_-, and S_1_-state lifetime measurements provide a powerful tool for studying the efficiency of PSII complexes under various conditions, in different species or mutants. By comparing the data obtained here with *C. merolae* thylakoids to our previous spinach data, we have demonstrated that the redox potential of *Q*_A_^−^/*Q*_A_ plays only a minor role for the miss parameter, but instead mostly affects the S_*i*_-state lifetimes. This is likely due to the increased energy gap between the *Q*_A_^−^/*Q*_A_ and Pheo^−^/Pheo redox pairs, which increases the barrier for the acceptor side electrons for recombination with the holes on the donor side, which occurs via the pheophytin cofactor (see Fig. 2). In addition, our data support the notion that the S_2_ → S_3_ transition is, at neutral pH, the least efficient step during the oxidation of water to molecular oxygen in the water-splitting enzyme, possibly due to the significant conformational changes occurring during the S_2_ → S_3_ transition, which include the binding of one water molecule (Suzuki et al. 2008; Siegbahn 2009; Capone et al. 2016; Retegan et al. 2016; Ugur et al. 2016; Kim and Debus 2017; Suga et al. 2017).
